# 4-[(2-Carb­oxy­eth­yl)amino]­benzoic acid monohydrate

**DOI:** 10.1107/S1600536812009518

**Published:** 2012-03-17

**Authors:** Chunman Jia, Shangwen Chen, Wenbing Yuan

**Affiliations:** a Key Laboratory of Tropical Biological Resources of the Ministry of Education, Hainan University, Haikou 570228, People’s Republic of China

## Abstract

In the title compound, C_10_H_11_NO_4_·H_2_O, the carboxyl group is twisted at a dihedral angle of 6.1 (3)° with respect to the benzene ring. In the crystal, the organic mol­ecules are linked by pairs of O—H⋯O hydrogen bonds involving both carboxyl groups, forming zigzag chains propagating along the *b*-axis direction. The water mol­ecules form [100] chains linked by O—H⋯O hydrogen bonds. The organic mol­ecule and water chains are cross-linked by N—H⋯O_water_ and O_water_—H⋯O hydrogen bonds, generating (001) sheets.

## Related literature

For synthetic background, see: Kurd & Hayao (1952 ▶); Yong *et al.* (2004 ▶).
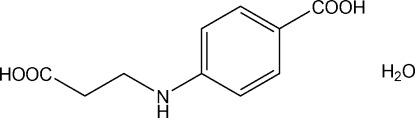


## Experimental

### 

#### Crystal data

C_10_H_11_NO_4_·H_2_O
*M* *_r_* = 227.21
Orthorhombic, 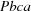

*a* = 4.9387 (19) Å
*b* = 19.700 (7) Å
*c* = 21.616 (8) Å
*V* = 2103.1 (14) Å^3^
*Z* = 8
Mo *K*α radiation
μ = 0.12 mm^−1^
*T* = 298 K
0.50 × 0.20 × 0.10 mm

#### Data collection

Rigaku AFC-7S Mercury diffractometer
Absorption correction: multi-scan (*CrystalClear*; Rigaku, 2005 ▶) *T*_min_ = 0.944, *T*_max_ = 0.989
15116 measured reflections
2401 independent reflections
2059 reflections with *I* > 2σ(*I*)
*R*_int_ = 0.037

#### Refinement

*R*[*F* ^2^ > 2σ(*F* ^2^)] = 0.056
*wR*(*F* ^2^) = 0.167
*S* = 1.09
2401 reflections
146 parameters
H-atom parameters constrained
Δρ_max_ = 0.21 e Å^−3^
Δρ_min_ = −0.29 e Å^−3^

### 

Data collection: *CrystalClear* (Rigaku, 2005 ▶); cell refinement: *CrystalClear*; data reduction: *CrystalClear*; program(s) used to solve structure: *SHELXS97* (Sheldrick, 2008 ▶); program(s) used to refine structure: *SHELXL97* (Sheldrick, 2008 ▶); molecular graphics: *SHELXTL* (Sheldrick, 2008 ▶); software used to prepare material for publication: *SHELXTL*.

## Supplementary Material

Crystal structure: contains datablock(s) I, global. DOI: 10.1107/S1600536812009518/hb6663sup1.cif

Structure factors: contains datablock(s) I. DOI: 10.1107/S1600536812009518/hb6663Isup2.hkl

Supplementary material file. DOI: 10.1107/S1600536812009518/hb6663Isup3.cml

Additional supplementary materials:  crystallographic information; 3D view; checkCIF report

## Figures and Tables

**Table 1:** Hydrogen-bond geometry (Å, °)

*D*—H⋯*A*	*D*—H	H⋯*A*	*D*⋯*A*	*D*—H⋯*A*
N7—H7⋯O5	0.97	2.04	3.009 (2)	175
O2—H2⋯O4^i^	0.95	1.74	2.662 (2)	165
O3—H1⋯O1^ii^	1.04	1.63	2.622 (2)	159
O5—H5*A*⋯O2^iii^	0.88	2.44	3.103 (2)	133
O5—H5*A*⋯O4^iv^	0.88	2.52	3.129 (2)	127
O5—H5*B*⋯O5^iv^	0.91	2.01	2.890 (2)	163

Symmetry codes: (i) 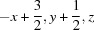; (ii) 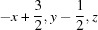; (iii) 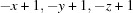; (iv) 
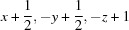
.

**Fig. 1.**
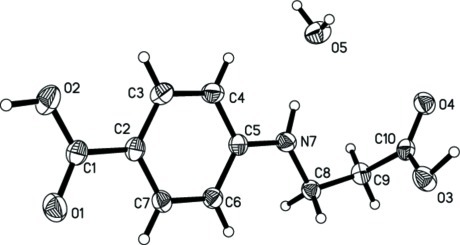
Ellipsoid plot.

**Fig. 2.**
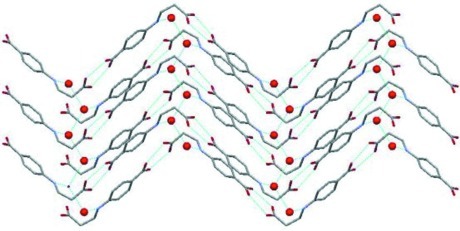
Packing diagram.

## supplementary crystallographic information

### Comment

The N-substitute β-alanine derivatives, as chelating or bridging muti-caboxylate ligands, have been applied to synthesis various novel metal-organic coordination polymers(Yong *et al.* 2004). It was firstly reported the reaction of propiolactone with aniline derivatives to synthesis the title compound I (Kurd & Hayao 1952). The molecular structure of the title compound I is shown in Fig. 1.

In the compound I, the carboxyl group attached to the benzene ring is twisted from its plane at 6.05 (29)°. For this compound, there are four main hydrongen bonds, and their distances are 2.622(O3—H1···O1^i^), 2.662(O2—H2···O4^iv^), 2.889(O5—H5B···O5^iii^) and 3.009(N7—H7···O5)Å, respectively. Throngh the intermolecular hydrogen bonds(O3—H1···O1^i^, O2—H2···O4^iv^) in carboxyl groups, generating *R*_2_^2^(8) loops, two title compound I moleculars connect together and form a *Z*-type one-dimensional polymer. The crystal waters form the water chains in the structure *via* hydrogen bonds(O5—H5B···O5^iii^). Morever, there are hydrogen bonds N7—H7···O5 between the crystal waters and compounds. As a result, all these hydrogen bonds connect the title compond I molculars into a layer (Fig. 2).

Symmetry codes: (i) -*x* + 3/2, *y* - 1/2, *z*; (ii) -*x* + 1, -*y* + 1, -*z* + 1; (iii) *x* + 1/2, -*y* + 1/2, -*z* + 1; (iv) -*x* + 3/2, *y* + 1/2, *z*.

### Experimental

The title compound (I) was prepared using a slightly modified published procedure (Yong *et al.* 2004). A solution of KOH (11.2 g, 0.2 mol) in water (60 ml) was added dropwise to a solution of 3-chloropropanonic acid (10.9 g, 0.1 mol) in water (50 ml). To the resulting alkline solution, *p*-aminobenzonic(13.7 g, 0.1 mol) was slowly added, and the mixture was refluxed for 36 h. The solution was filtered and cooled to room temperature, then acidified with HCl solution until the desired white precipitated, which were collected by filtration. Pure compound (I) was obstained by crystallizing from methanol. Colourless blocks of (I) were obstained by slow evaporation of methanol solution.

### Refinement

H atoms were positioned geometrically (C–H = 0.95–0.99 Å) and allowed to ride on their parent atoms with *U*_iso_(H) = 1.2 *U*_eq_(C).

### Crystal data

**Table:**

C_10_H_11_NO_4_·H_2_O	*F*(000) = 960
*M**_r_* = 227.21	*D*_x_ = 1.435 Mg m^−^^3^
Orthorhombic, *P**b**c**a*	Mo *K*α radiation, λ = 0.71073 Å
Hall symbol: -P 2ac 2ab	Cell parameters from 4011 reflections
*a* = 4.9387 (19) Å	θ = 2.3–27.4°
*b* = 19.700 (7) Å	µ = 0.12 mm^−^^1^
*c* = 21.616 (8) Å	*T* = 298 K
*V* = 2103.1 (14) Å^3^	Prism, colourless
*Z* = 8	0.50 × 0.20 × 0.10 mm

### Data collection

**Table:**

Rigaku AFC-7S Mercury diffractometer	2401 independent reflections
Radiation source: Rotating Anode	2059 reflections with *I* > 2σ(*I*)
Confocal monochromator	*R*_int_ = 0.037
Detector resolution: 28.5714 pixels mm^-1^	θ_max_ = 27.5°, θ_min_ = 2.8°
CCD_Profile_fitting scans	*h* = −6→6
Absorption correction: multi-scan (*CrystalClear*; Rigaku, 2005)	*k* = −25→17
*T*_min_ = 0.944, *T*_max_ = 0.989	*l* = −27→28
15116 measured reflections	

### Refinement

**Table:**

Refinement on *F*^2^	Primary atom site location: structure-invariant direct methods
Least-squares matrix: full	Secondary atom site location: difference Fourier map
*R*[*F*^2^ > 2σ(*F*^2^)] = 0.056	Hydrogen site location: inferred from neighbouring sites
*wR*(*F*^2^) = 0.167	H-atom parameters constrained
*S* = 1.09	*w* = 1/[σ^2^(*F*_o_^2^) + (0.0855*P*)^2^ + 0.7233*P*] where *P* = (*F*_o_^2^ + 2*F*_c_^2^)/3
2401 reflections	(Δ/σ)_max_ < 0.001
146 parameters	Δρ_max_ = 0.21 e Å^−^^3^
0 restraints	Δρ_min_ = −0.29 e Å^−^^3^

### Special details

**Table:**

Geometry. All e.s.d.'s (except the e.s.d. in the dihedral angle between two l.s. planes) are estimated using the full covariance matrix. The cell e.s.d.'s are taken into account individually in the estimation of e.s.d.'s in distances, angles and torsion angles; correlations between e.s.d.'s in cell parameters are only used when they are defined by crystal symmetry. An approximate (isotropic) treatment of cell e.s.d.'s is used for estimating e.s.d.'s involving l.s. planes.
Refinement. Refinement of *F*^2^ against ALL reflections. The weighted *R*-factor *wR* and goodness of fit *S* are based on *F*^2^, conventional *R*-factors *R* are based on *F*, with *F* set to zero for negative *F*^2^. The threshold expression of *F*^2^ > σ(*F*^2^) is used only for calculating *R*-factors(gt) *etc*. and is not relevant to the choice of reflections for refinement. *R*-factors based on *F*^2^ are statistically about twice as large as those based on *F*, and *R*- factors based on ALL data will be even larger.

### Fractional atomic coordinates and isotropic or equivalent isotropic displacement parameters (Å^2^)

**Table:**

	*x*	*y*	*z*	*U*_iso_*/*U*_eq_	
O4	0.0461 (3)	0.17797 (7)	0.60257 (7)	0.0513 (4)	
O3	0.2084 (3)	0.20433 (7)	0.69553 (7)	0.0560 (4)	
O1	0.9408 (3)	0.60527 (7)	0.68389 (8)	0.0552 (4)	
C2	0.7123 (4)	0.51980 (9)	0.62835 (9)	0.0386 (4)	
O2	1.0540 (3)	0.58943 (8)	0.58500 (7)	0.0570 (4)	
C8	−0.0056 (4)	0.33809 (9)	0.67078 (9)	0.0397 (4)	
H8A	−0.1384	0.3739	0.6760	0.048*	
H8B	0.0950	0.3338	0.7091	0.048*	
C7	0.5435 (4)	0.50574 (9)	0.67792 (9)	0.0381 (4)	
H6	0.5525	0.5327	0.7132	0.046*	
N7	0.1779 (3)	0.35565 (8)	0.62101 (7)	0.0420 (4)	
C6	0.3618 (4)	0.45234 (9)	0.67594 (9)	0.0382 (4)	
H5	0.2502	0.4438	0.7097	0.046*	
C5	0.3452 (4)	0.41113 (9)	0.62324 (9)	0.0369 (4)	
C10	0.0490 (4)	0.21407 (9)	0.65073 (9)	0.0395 (4)	
C9	−0.1486 (4)	0.27183 (10)	0.65677 (10)	0.0427 (5)	
H9A	−0.2760	0.2618	0.6897	0.051*	
H9B	−0.2500	0.2764	0.6186	0.051*	
C4	0.5114 (4)	0.42647 (10)	0.57240 (9)	0.0447 (5)	
H4	0.4999	0.4004	0.5366	0.054*	
C3	0.6909 (4)	0.47976 (10)	0.57507 (9)	0.0450 (5)	
H3	0.7995	0.4893	0.5410	0.054*	
C1	0.9125 (4)	0.57462 (9)	0.63300 (9)	0.0403 (4)	
O5	0.1077 (4)	0.28799 (9)	0.49724 (8)	0.0680 (5)	
H1	0.3415	0.1673	0.6803	0.082*	
H5A	0.1216	0.3062	0.4601	0.082*	
H5B	0.2810	0.2725	0.4992	0.082*	
H2	1.1723	0.6267	0.5907	0.082*	
H7	0.1518	0.3363	0.5801	0.059 (7)*	

### Atomic displacement parameters (Å^2^)

**Table:**

	*U*^11^	*U*^22^	*U*^33^	*U*^12^	*U*^13^	*U*^23^
O4	0.0567 (9)	0.0465 (8)	0.0507 (9)	0.0084 (6)	−0.0045 (7)	−0.0030 (6)
O3	0.0587 (9)	0.0514 (8)	0.0578 (10)	0.0159 (7)	−0.0138 (7)	−0.0084 (7)
O1	0.0605 (10)	0.0455 (8)	0.0597 (10)	−0.0115 (7)	−0.0070 (7)	−0.0042 (7)
C2	0.0365 (9)	0.0352 (9)	0.0441 (10)	−0.0001 (7)	−0.0031 (7)	0.0057 (7)
O2	0.0542 (9)	0.0586 (9)	0.0583 (10)	−0.0170 (7)	−0.0009 (7)	0.0130 (7)
C8	0.0384 (9)	0.0364 (9)	0.0444 (10)	0.0031 (7)	0.0034 (8)	−0.0010 (7)
C7	0.0408 (9)	0.0322 (9)	0.0412 (10)	0.0028 (7)	−0.0032 (8)	−0.0012 (7)
N7	0.0443 (9)	0.0409 (8)	0.0407 (9)	−0.0052 (7)	0.0030 (7)	−0.0046 (6)
C6	0.0381 (9)	0.0366 (9)	0.0401 (10)	0.0021 (7)	0.0020 (7)	0.0007 (7)
C5	0.0354 (9)	0.0340 (8)	0.0414 (10)	0.0026 (7)	−0.0020 (7)	0.0020 (7)
C10	0.0386 (9)	0.0339 (9)	0.0460 (11)	−0.0030 (7)	0.0019 (8)	0.0044 (7)
C9	0.0344 (9)	0.0407 (10)	0.0530 (11)	−0.0004 (7)	0.0003 (8)	−0.0001 (8)
C4	0.0487 (11)	0.0481 (11)	0.0372 (10)	−0.0052 (9)	0.0023 (8)	−0.0043 (8)
C3	0.0447 (10)	0.0510 (11)	0.0392 (10)	−0.0048 (8)	0.0029 (8)	0.0049 (8)
C1	0.0394 (9)	0.0375 (9)	0.0441 (10)	0.0004 (7)	−0.0030 (8)	0.0065 (8)
O5	0.0777 (12)	0.0765 (12)	0.0498 (10)	−0.0008 (10)	0.0038 (8)	0.0037 (8)

### Geometric parameters (Å, º)

**Table:**

O4—C10	1.261 (2)	C7—H6	0.9300
O3—C10	1.263 (2)	N7—C5	1.371 (2)
O3—H1	1.0351	N7—H7	0.9720
O1—C1	1.262 (2)	C6—C5	1.401 (3)
C2—C7	1.386 (3)	C6—H5	0.9300
C2—C3	1.400 (3)	C5—C4	1.404 (3)
C2—C1	1.468 (3)	C10—C9	1.505 (3)
O2—C1	1.284 (2)	C9—H9A	0.9700
O2—H2	0.9458	C9—H9B	0.9700
C8—N7	1.448 (3)	C4—C3	1.375 (3)
C8—C9	1.515 (3)	C4—H4	0.9300
C8—H8A	0.9700	C3—H3	0.9300
C8—H8B	0.9700	O5—H5A	0.8817
C7—C6	1.383 (3)	O5—H5B	0.9095
			
C10—O3—H1	105.0	N7—C5—C4	119.75 (17)
C7—C2—C3	118.57 (17)	C6—C5—C4	118.51 (17)
C7—C2—C1	119.96 (17)	O4—C10—O3	123.67 (18)
C3—C2—C1	121.45 (17)	O4—C10—C9	119.40 (17)
C1—O2—H2	114.0	O3—C10—C9	116.92 (17)
N7—C8—C9	110.43 (16)	C10—C9—C8	111.50 (15)
N7—C8—H8A	109.6	C10—C9—H9A	109.3
C9—C8—H8A	109.6	C8—C9—H9A	109.3
N7—C8—H8B	109.6	C10—C9—H9B	109.3
C9—C8—H8B	109.6	C8—C9—H9B	109.3
H8A—C8—H8B	108.1	H9A—C9—H9B	108.0
C6—C7—C2	121.22 (17)	C3—C4—C5	120.54 (18)
C6—C7—H6	119.4	C3—C4—H4	119.7
C2—C7—H6	119.4	C5—C4—H4	119.7
C5—N7—C8	122.78 (15)	C4—C3—C2	120.87 (18)
C5—N7—H7	115.1	C4—C3—H3	119.6
C8—N7—H7	119.9	C2—C3—H3	119.6
C7—C6—C5	120.24 (17)	O1—C1—O2	122.35 (18)
C7—C6—H5	119.9	O1—C1—C2	119.08 (17)
C5—C6—H5	119.9	O2—C1—C2	118.57 (17)
N7—C5—C6	121.71 (17)	H5A—O5—H5B	96.1

### Hydrogen-bond geometry (Å, º)

**Table:**

*D*—H···*A*	*D*—H	H···*A*	*D*···*A*	*D*—H···*A*
N7—H7···O5	0.97	2.04	3.009 (2)	175
O2—H2···O4^i^	0.95	1.74	2.662 (2)	165
O3—H1···O1^ii^	1.04	1.63	2.622 (2)	159
O5—H5*A*···O2^iii^	0.88	2.44	3.103 (2)	133
O5—H5*A*···O4^iv^	0.88	2.52	3.129 (2)	127
O5—H5*B*···O5^iv^	0.91	2.01	2.890 (2)	163

Symmetry codes: (i) −*x*+3/2, *y*+1/2, *z*; (ii) −*x*+3/2, *y*−1/2, *z*; (iii) −*x*+1, −*y*+1, −*z*+1; (iv) *x*+1/2, −*y*+1/2, −*z*+1.
